# Sensor Node Activation Using Bat Algorithm for Connected Target Coverage in WSNs

**DOI:** 10.3390/s20133733

**Published:** 2020-07-03

**Authors:** Jaemin Kim, Younghwan Yoo

**Affiliations:** School of Electrical and Computer Engineering, Pusan National University, Busan 46241, Korea; repushed@gmail.com

**Keywords:** wireless sensor networks, probabilistic sensing model, connected target coverage, bat algorithm, sensor node activation

## Abstract

This paper proposes a sensor node activation method using the nature-inspired algorithm (NIA) for the target coverage problem. The NIAs have been used to solve various optimization problems. This paper formulates the sensor target coverage problem into an object function and solves it with an NIA, specifically, the bat algorithm (BA). Although this is not the first attempt to use the BA for the coverage problem, the proposed method introduces a new concept called bat couple which consists of two bats. One bat finds sensor nodes that need to be activated for sensing, and the other finds nodes for data forwarding from active sensor nodes to a sink. Thanks to the bat couple, the proposed method can ensure connectivity from active sensor nodes to a sink through at least one communication path, focusing on the energy efficiency. In addition, unlike other methods the proposed method considers a practical feature of sensing: The detection probability of sensors decreases as the distance from the target increases. Other methods assume the binary model where the success of target detection entirely depends on whether a target is within the threshold distance from the sensor or not. Our method utilizes the probabilistic sensing model instead of the binary model. Simulation results show that the proposed method outperforms others in terms of the network lifetime.

## 1. Introduction

Recently, wireless sensor networks (WSNs) have been used in various fields, such as surveillance, military tracking, disaster management, biomedical health monitoring, and targeting systems. WSNs consist of many small sensor nodes which are densely deployed in a region. The sensor nodes work together to monitor the region to obtain environmental data, and send their data to a sink node (also called a base station). The sensor nodes have hardware constraints on computing, storage, and communication capacity. They also have a limited battery level, and it is difficult to replace or recharge their battery. Therefore, the energy-efficient strategy controlling sensor nodes is the most important in WSNs [1].

Some studies have been interested in a sensing coverage problem of WSNs. The sensing coverage problem means how completely sensors monitor the interested region (or points). The coverage has three types: area coverage, target (point) coverage, and barrier coverage. As the names suggest, the goal of the area coverage is to monitor an area, and the target coverage is to cover a set of points. The barrier coverage is to detect that intruders cross borders or penetrate protected areas [2]. In order to provide the coverage effectively, it is important to properly deploy nodes in the region of interest. However, in harsh environment such as battlefields, underwater, and disaster areas, nodes may not be placed at desired position. Instead, sensors are usually placed at random position in the region. In this situation, the network lifetime can be maximized by adopting an efficient sensor node activation scheme that turns on the minimum number of sensor nodes required to completely monitor an interested region (or targets) at any time [3].

When designing a node activation method, the most important consideration is the connectivity, which means that at least one path must exist between an active sensor and the sink node [4,5]. Since each node may not be able to communicate directly with the sink node due to the limited communication range, packets should be forwarded along multi-hop paths through other nodes. If the nodes observing targets are not directly connected to the sink node, additional sensor nodes need to be activated for relaying operation. These relay nodes are only responsible for forwarding data to intermediate or sink nodes, not for sensing targets.

It is also recommended to consider the practical characteristics of a sensor unit for the more energy-efficient sensor activation method. The real sensing capability of sensors (such as sonar, heat, force, etc.) depends on the distance from a target (or a region) being monitored. However, most methods [6,7,8,9,10,11] adopted the binary sensing model. Under the binary sensing model, the desired targets (or regions) are said to be covered if they are within the sensing area of the sensors. This sensing model facilitates the analysis and helps understand the coverage problem, but it is only a coarse approximation to the practical sensing model [12]. Therefore, the sensor activation methods should reflect the practical behavior of the sensor units.

Considering various realistic environment, it is so difficult to design a sensor activation method since traditional deterministic methods or algorithms are not suitable for solving a large number of problems simultaneously [13]. Therefore, a good candidate method for this is a nature-inspired algorithm (NIA). NIAs imitating natural phenomena have been used in various fields, including to solve various types of coverage problems in WSNs [14,15,16,17,18,19,20,21]. However, in our knowledge, there is no NIA-based node activation method which considers both the probabilistic sensing model and connectivity for the target coverage problem. A good design of a NIA-based sensor activation method that controls the state of sensor nodes can maximize the network lifetime.

This paper proposes an NIA-based node activation method, focusing on the target coverage problem with considering practical features such as sensing capability of sensors and connectivity between a sensor and a sink node. To resolve the complex problem considering the practical features as well, we employ the bat algorithm (BA) inspired by the behavior of bats finding food. For solving both the coverage problem and the connectivity problem together, the BA is enhanced to use a new concept of bat couple. Out of a couple of bats, one bat finds the sensor nodes that play a sensing role, and the other finds the nodes that forward data of the sensing node to a sink. The greedy method is used to set the initial position of bats, since it can make the problem to quickly converge to an optimal solution, compared with the random position initialization.

The contributions of this paper are summarized as follows: (a) Many researchers have proposed sensor activation methods that use nature-inspired algorithm (NIA). Their computation speed, however, is very slow. In contrast, our method relatively has faster computation speed because an initialization technique in BA is used. (b) Additionally, most methods with NIA do not consider the connectivity between a sink and active sensors. Our method considers connectivity with a sink node by introducing the bat couple concept.

The remainder of this paper is organized as follows: Section 2 introduces related works of the target coverage problem and the BA’s characteristics. Section 3 describes an overview of the network model that we assume in this paper. In Section 4, we briefly explain the overall step of the conventional BA. Section 5 explains the proposed method based on the BA in detail and the performance is evaluated through simulation in Section 6. Finally, Section 7 presents the conclusion and future works.

## 2. Related Work

### 2.1. Coverage Problem

Many schemes to ensure sensing coverage have tried to maximize network lifetime as well. One of them is the study that properly activates and deactivates sensor nodes so that the area can be fully covered with the minimum energy consumption of sensor nodes. In the active state, a sensor node observes a target (or area) or is in the Tx, Rx, or Idle mode for communication, while a node in the inactive state is in the sleep mode. Therefore, it is necessary to activate the fewest possible number of sensor nodes at every moment in order to minimize the overall energy consumption of the network.

Various studies assume the different types of sensing characteristics in their sensing models. In practice, sensing accuracy depends on the distance between sensor and target, because the signal strength (such as radios, acoustics, and seismic) decreases as the distance increases [22]. This sensing model, where the detection probability of sensors decreases as the distance from targets increases, is called probabilistic sensing model [23]. However, many previous researches assumed the binary sensing model where the success of the detection depends entirely on whether the distance is within the threshold:

(1) $$\lambda_{i}\left(j\right)=\left\{\begin{array}{c} 1,\;\mathrm{if}\;d_{ij}\leq r\\ 0,\;\mathrm{otherwise} \end{array}\right.$$

where $d_{ij}$ is the distance between sensor *i* and point of interest (PoI; target) *j* and *r* is the detection range (threshold) of the sensor *i*. Thus, if $d_{ij}$ is less than *r*, it can say that the sensor *i* can surely detect the PoI *j*.

**Figure 1:**
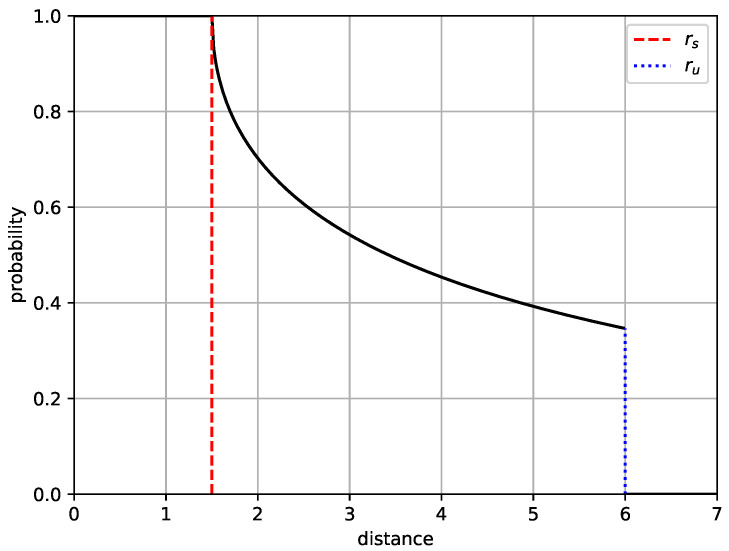
Example of the probabilistic sensing model with $r_{s}=1.5$ m, $r_{u}=6$ m, k=0.5 and q=0.5.

On the contrary, in the probabilistic sensing model, as the distance between a sensor node and a PoI increases, the sensor detection probability decreases exponentially [24]. Let $\lambda_{i}\left(j\right)$ denote the detection probability of sensor *i* about events at PoI *j*. As introduced in [24], the detection probability of sensors is formulated as:

(2) $$\lambda_{i}\left(j\right)=\left\{\begin{array}{cc} 1, & \mathrm{if}\;d_{ij}\leq r_{s}\\ \exp^{-k{\left(d_{ij}-r_{s}\right)}^{q}}, & \mathrm{if}\;r_{s}<d_{ij}\leq r_{u}\\ 0, & \mathrm{otherwise} \end{array}\right.$$

where $d_{ij}$ is the distance between node *i* and PoI *j*. If PoI *j* is closer than the distance $r_{s}$, the event at PoI *j* can be surely detected. If $d_{ij}$ is greater than $r_{s}$ and less than $r_{u}$, the detection probability decreases exponentially with the increase of distance $d_{ij}$. If $d_{ij}$ is greater than $r_{u}$, the probability is set to zero. The parameters *k*, *q*, $r_{s}$ and $r_{u}$ depend on the type of physical sensors and environment factors [24]. Figure 1 shows an example of probabilistic sensing model with $r_{s}=1.5$ m, $r_{u}=6$ m, k=0.5 and q=0.5. The *X*-axis represents the distance between a sensor and a PoI, and the *Y*-axis represents the detection probability.

Some PoIs may be covered by more than one sensor nodes. The detection probability for this kind of PoI can be calculated using the product of the individual probability of all sensors [22]. If $S_{j}$ is the set of sensor nodes that can cover PoI *j*, the cumulative detection probability $\lambda_{S_{j}}\left(j\right)$ can be obtained as:

(3) $$\lambda_{S_{j}}\left(j\right)=1-\prod_{i\in S_{j}}\left(1-\lambda_{i}\left(j\right)\right)$$

If $\lambda_{S_{j}}\left(j\right)\geq \epsilon$, where ϵ is the minimum threshold of the detection probability, we can say that PoI *j* is covered.

The optimal sensor activation scheme using the probabilistic sensing model is known as an NP-hard problem, thus the states of all sensor nodes cannot be determined in the exhaustive manner. Instead, heuristic algorithms have been used over the decades. In [25], the target coverage problem was formulated as a binary integer programming (BIP) and the authors proposed four methods to solve the BIP: linear programming relaxation and rounding, greedy heuristic, greedy approximation, and Lagrangian heuristic. The simulation results showed that the greedy heuristic method has the best performance, considering detection accuracy and computation time together. On the other hand, research in [26] proposed another greedy algorithm: First, it computes the contribution (weight) of each sensor according to how many targets can be covered by the sensor. Then the sensors are activated one by one in the order of higher contribution until all targets are covered. The authors evaluated the performance in four different sensing models (one binary model and three probabilistic sensing models).

The nature-inspired algorithms (NIAs) imitate nature in order to solve many complex problems, including the sensor node activation for target coverage with the probabilistic sensing model. In [19], two NIAs, ant colony optimization (ACO) and particle swarm optimization (PSO), were utilized, and their method outperformed the greedy method in [25] in terms of network lifetime. The ACO is the method mimicking how ants use pheromone to find food. In [20], for the coverage problem, the basic ACO was enhanced to use three types of pheromones (one local pheromone and two global pheromones), so this method is called Three Pheromones ACO (TPACO). The local pheromone helps an ant construct a coverage set with fewer sensors. As to the two global pheromones, one is to minimize the number of required active sensors per target and the other is to form a sensor set by using the former pheromone. Research [21] suggested a Jenga-inspired optimization algorithm (JOA) that mimics the well-known board game Jenga. Initially, a tower is built of blocks, each block indicating a sensor. Virtual players take turns removing one block of the tower according to a rule, and a sensor corresponding to the removed block goes to an inactive state. The lowest cost tower under the condition that it does not collapse is referred as the optimal solution at the time. JOA has faster convergence time than ACO, PSO, and TPACO.

Some studies considered connectivity as well as target coverage with the probabilistic sensing model. The paper [27] proposed a greedy method called localized coverage quality algorithm (LoCQAl) that uses three categories of sensors. Nodes in the first category make up the connected dominating set (CDS), and nodes in the second category cover multiple targets. The remaining sensors fall into the third category. Nodes in each category are turned on or off according to a rule. LoCQAl is applied to mobile sensor networks using two redeployment schemes where sensor nodes move to cover all targets with almost the same number of nodes. In the simulation result, the algorithm in static sensor networks has poor network lifetime, but it shows better performance than virtual force algorithm [28] in mobile WSNs in terms of network lifetime and computation overhead. In [29], probabilistic sensor cover algorithm (PSCA) was proposed for both target coverage and connectivity. PSCA uses the depth-first search (DFS) to find candidate cover sets (CCSs) each of which includes nodes needed to cover all targets. A greedy method is used to choose the best cover set from CCSs and the Steiner tree-based algorithm finds the nodes that will act as the relay nodes for connectivity. The authors evaluated their method in comparison with LoCQAl in terms of network energy consumption and the number of active sensors. Simulation results showed that PSCA reduced the both metrics to about 50% of LoCQAl.

### 2.2. Bat Algorithm

Over the last few decades, many nature-inspired algorithms (NIAs) have been proposed to solve various optimization problems which mainly find the minimum or maximum value of cost functions. In particular, although the bat algorithm is a relatively recent method [30], it has attracted the attention of many researchers. The BA is inspired by the echolocation behavior of microbats that use sonar for food searching and obstacle detection. The virtual bats in the BA find a solution of a problem, by behaving like real bats. They fly randomly with an arbitrary velocity, frequency and loudness to search for foods (solutions). They can adjust the frequency of their emitted pulses according to the proximity of their target. The general steps of this algorithm are described in Section 4. The BA shows better performance compared with other NIAs such as genetic algorithm and particle swarm optimization [30,31]. In addition, due to the simplicity and ease of implementation, many researchers have proposed the modified BAs to solve various real-world problems [32].

In [33], the authors proposed a chaotic bat algorithm (CBA) for solving the economic dispatch (ED) problem which aims to allocate the optimal generation value to minimize the cost in electric power system. The CBA uses a chaotic map or sequence to adjust loudness of each bat. The results showed that the CBA can cope with high dimensional ED problems with several constraints.

The paper [34] proposed the bat algorithm with mutation (BAM) which utilizes the mutation operation of differential evolution (DE) helping bats find a global solution faster. The BAM is used for image matching to discover the position of an object in the original image when the corresponding two grey scale images are given. The BAM outperforms DE, BA, and stud genetic algorithm in terms of time complexity.

The standard BA is suitable to solve the problem with continuous real search space, but it cannot be applied to the problem with discrete binary search spaces. In [35], the authors proposed the binary bat algorithm (BBA) to solve binary problems. The BBA has artificial bats navigating and hunting in binary search spaces by changing their positions from “0" to “1" and vice versa. The simulation results showed the superiority of the BBA to the binary PSO and genetic algorithm. The results also proved that the BBA can be effective for optical buffer design in optical engineering.

In addition, the BA has demonstrated its effectiveness in the fields such as visual tracking [36], brushless DC motor design [37], feature selection [38], multiprocessor scheduling [39], detection of malicious code variants [40], image classification [41], and so on. Furthermore, the BA has the fast convergence speed to a global solution and is suitable for large-scaled problems [35]. Therefore, the BA has potential to solve many different problems on WSNs with many sensors.

## 3. Problem Description

As mentioned earlier, there are three types of coverage problems: area coverage, target coverage, and barrier coverage. Among them, this paper handles the target coverage where points of interest (PoIs; target positions) are covered by active sensor nodes. In addition, the connectivity from active sensor nodes to the sink is guaranteed.

The sensor network in this paper has the following properties:

- All sensor nodes have the same hardware constraints and battery energy level.
- Nodes are immobile and uniformly deployed in a 2D euclidean plane. Areas of interest are difficult for people to access, e.g., disaster areas and military areas.
- All sensors are homogeneous and omni-directional sensors that can observe 360-degrees at the same time.
- All sensor nodes know their location by using GPS or some localization techniques [42].
- In each timeslot, each sensor node is in one of three modes: sensing mode, relaying mode, sleep mode. Nodes in the sensing mode, called *sensing node*, sense PoIs and communicate with other nodes in order to send their data to sink node. Nodes in the relay mode, called *relay node*, only forward data of sensing nodes toward the sink. Finally, nodes in the sleep mode, called *sleeping node*, just sleep, not doing anything.
- A sink node, or a base station, is located at the edge of the region of interest and periodically receives sensor data from monitored PoIs at fixed positions.
- The sink node determines and tells which sensor node to activate. Our method is a centralized computation model.
- In pursuit of a pragmatic approach, we adopt a probabilistic sensor detection model.

We formulated the target coverage as an object function. Based on the function in [25] that only considers sensing coverage, we enhanced it to ensure the connectivity as well:

(4) $$f\left(S_{s},S_{r}\right)=\sum_{i\in S_{s}}\left\{\left(C_{s}+C_{r}\right)\times w_{i}\right\}+\sum_{i\in S_{r}}\left(C_{r}\times w_{i}\right)$$

where $S_{s}$ and $S_{r}$ are the set of sensing nodes and the set of relay nodes in a timeslot respectively. $C_{s}$ is the energy cost for a sensing node to detect targets and $C_{r}$ is the energy cost for a packet relay to an adjacent node. A sensing node consumes the energy of $\left(C_{s}+C_{r}\right)$ because it has to not only sense targets but also send the sensed data to its neighbor. A relay node consumes only the energy of $C_{r}$. Note that our object function $f(S_{s},S_{r})$ is not the simple sum of energy cost but a weighted sum.

We make the value of energy exponentially increase as the amount of remaining energy decreases by using two variables $e_{i}$ and κ: $e_{i}$ is the remaining energy of sensor node *i* and κ is a constant in (0,1] which is related to the characteristics of the sensor:

(5) $$w_{i}=\kappa^{e_{i}}$$

If κ is 1, then the weight *w* is 1 no matter how much energy is left in each sensor. On the other hand, if the value of κ is between 0 and 1, the weight reflects the residual energy of sensors. Therefore, a sensor which has low energy is hard to be added to the active sensor set ($S_{s}$ or $S_{r}$) because it has higher cost. As a result, the less $f(S_{s},S_{r})$ is, the more appropriate a set of $S_{s}$ and $S_{r}$ is as the final solution. So, the sink node determines the state of each sensor node at a timeslot by finding the set of sensor nodes with the least value of the object function.

## 4. Conventional Bat Algorithm

It is difficult to find the solution for the cost function *f* because it is an NP-Hard problem [19]. As mentioned in Section 2.2, the BA has the fast convergence speed to a global solution and is suitable for large-scaled problems. Considering such characteristics, we propose a sensor activation method based on BA because the sensing coverage problem assumes large-scale network environment with many sensor nodes.

Algorithm 1 presents the pseudo code of the BA. The BA works similarly to how real bats find foods. Suppose a number of virtual bats are flying to find food. The position, velocity, and ultrasound frequency of each bat are stored in position vector x, velocity vector v, and frequency vector f, respectively. In every iteration, the sound frequency of each bat randomly changes first; then it changes the velocity; subsequently, the position of each bat is updated according to the velocity change. Finally, the updated position x is inputted to the cost function *f*, and f(x) represents the cost at that iteration. As a result, bats get closer and closer to food every iteration. This process can be expressed as Equations (6)–(8).

(6) $$x_{i}\left(t+1\right)=x_{i}\left(t\right)+v_{i}\left(t+1\right)$$

(7) $$v_{i}\left(t+1\right)=v_{i}\left(t\right)+\left(x_{i}-x_{*}\right)f_{i}$$

where $x_{*}$ is the best position among all bats at the *t*-th iteration. $x_{i}\left(t\right)$ and $v_{i}\left(t\right)$ are the position and the velocity of the *i*-th bat at time *t*; and $f_{i}$ is the frequency of bat *i*, which is computed as:

(8) $$f_{i}=F_{min}+\left(F_{max}-F_{min}\right)\alpha$$

where α is a random vector of the uniform distribution in [0,1]. $F_{max}$ and $F_{min}$ are the maximum and minimum frequency that a bat can emit.

In order to avoid the local optimum by testing more various solutions, the BA generates the new position of each bat using the random walk:

(9) $$x_{new}=x_{old}+\epsilon a\left(t\right)$$

Here, ϵ is a random number in [−1,1] and a(t) is the sound loudness emitted by a bat at the *t*-th iteration. In nature, the loudness usually decreases once a bat has found its prey, whereas the pulse emission rate increases [30]. Thus, the loudness $a_{i}$ and the pulse emission rate $r_{i}$ of the *i*-th bat are updated in every iteration as Equations (10) and (11).

(10) $$a_{i}\left(t+1\right)=\beta a_{i}\left(t\right)$$

(11) $$r_{i}\left(t+1\right)=r_{i}\left(0\right)\left[1-exp\left(-\gamma t\right)\right]$$

where β and γ are constants. β is similar to the cooling factor in the simulated annealing (SA). The loudness and emission rate are adjusted when a new best solution is discovered (Line 12 of Algorithm 1). This means that the bat gets closer to the optimal value [35].
**Algorithm 1** Pseudo code of the bat algorithm [30].1:Initialize the bat population $x_{i}$ (i=1,2,…,n) and $v_{i}$2:Define frequency $f_{i}$ and pulse rate $r_{p}$3:**while**t< Max number of iterations **do**4: Generate new solutions by adjusting frequency, updating velocity and positions [Equation (6) to (8)]5: **if** $rand>r_{i}$ **then**6:  Select a solution among the best solutions7:  Generate a local solution around the selected best solution8: **end if**9: Generate a new solution by flying randomly10: **if** $rand<a_{i}$ and $f\left(x_{i}\right)<f\left(x_{*}\right)$ **then**11:  Accept the new solution12:  Increase $r_{i}$ and reduce $a_{i}$13: **end if**14: Rank the bats and find the current best $x_{*}$15:**end while**

## 5. Proposed Method

We introduce a BA-based sensor node activation for the target coverage problem. The sink node uses the bat algorithm (BA) to find the minimum value of the objective function *f*. In the conventional BA, the value in each dimension of the bat’s position vector is a real number, but the function *f* is defined on discrete sets $\left(S_{s},S_{r}\right)$. Thus, it is too hard to solve the function by using the normal algorithm. We propose the modified bat algorithm which can solve the objective function *f*. Our algorithm is based on a binary bat algorithm [35] but has some differences. Unlike the binary BA, our BA suggests that two bats work together as a couple in order to provide the connectivity from active sensors to the sink as well as the requested level of target coverage.

### 5.1. Position Vector for a Bat Couple

We introduce a new concept called bat couple into the conventional BA. In the proposed BA, a couple of bats work as a team: one bat $B_{s}$ finds nodes that should play the sensing role, and the other bat $B_{r}$ finds nodes that should relay the data to the sink node. In case of the normal binary BA, the value in each dimension of each position vector is 0 or 1. Thus, this BA can divide the state of each node into two types (activated node or sleep node). Therefore, the normal binary BA is difficult to represent all states of each node because the number of each sensor node’s states is 3 (relay node, sensing node and sleep node). Instead, we use the bat couple to represent the all states.

**Figure 2:**
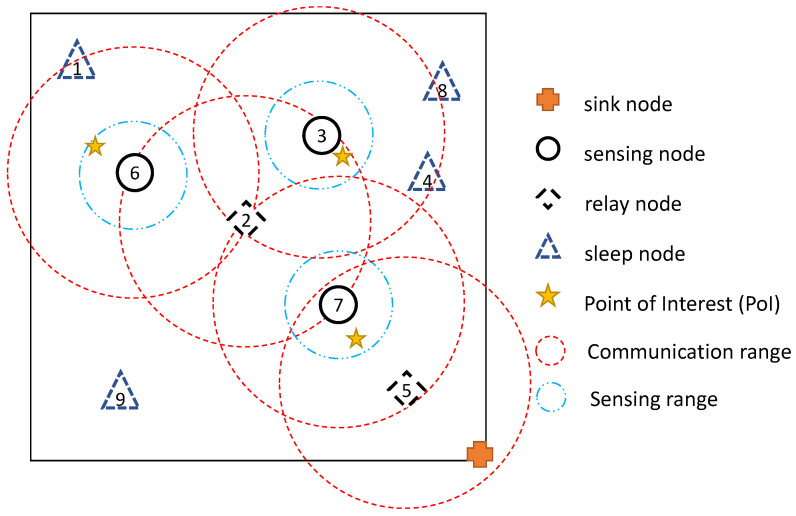
Example of network environment.

Accordingly, all the vectors for bat information such as position and velocity should be extended for two bats. Suppose there are nine sensors and three PoIs in the region of interest as Figure 2. The big and small dotted circles represent communication ranges and sensing ranges (The binary sensing range is assumed only for Figure 2. Our method basically uses the probabilistic sensing model in Euqation (2)) of sensor nodes respectively. Considering the ranges of each sensor, it may be the best case that nodes 3, 6, and 7 work as sensing nodes and nodes 2 and 5 work as relay nodes.

**Figure 3:**
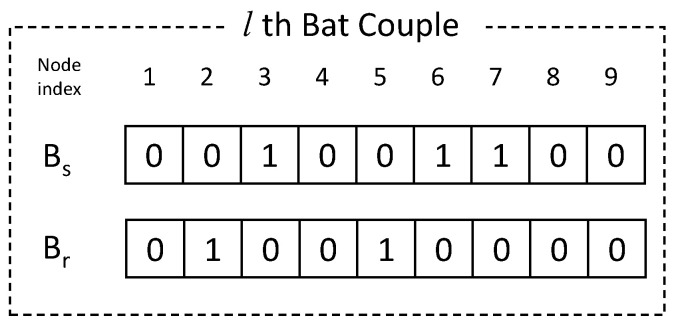
Position vector for a bat couple.

Figure 3 shows the position vector for a bat couple in the given network. Unlike the conventional BA, our vector has the position of two bats: $B_{s}$ to select sensing nodes and $B_{r}$ to select relay nodes. The position of each bat is represented by an *N*-dimensional vector, where *N* is the number of sensor nodes in the network; thus the position vector can be regarded as the set of reachability by the bat to each sensor node. Thus in Figure 3, we can think that each bat is at the position where it can see the nodes of which vector elements are set to 1. After finding the best position of bats based on Equation (4), nodes corresponding to the elements having the value of 1 in the vectors $B_{s}$ and $B_{r}$ are assigned the roles of the sensing node and the relay node, respectively.
**Algorithm 2** Proposed bat algorithm for connected coverage.1:Initialize the bat population v, f and x         // Algorithm 32:Find the best couple $x^{*}$ by evaluating position vector of all couples3:t←0 // *t*-**th iteration in main loop**4:**while**$t<N_{t}$**do**5: **for** *c* from 1 to $N_{c}$ **do**6:  Adjust frequency $f^{c}$ and velocity $v^{c}$7:  Update position vector $x^{tmp}$                  // Algorithm 48:  **if** $f\left(x^{tmp}\right)<f\left(x^{c}\right)$ and $Validate(x^{tmp}$) **then**9:   $x^{c}\leftarrow x^{tmp}$10:   **if** $f\left(x^{tmp}\right)<f\left(x^{*}\right)$ **then**11:    $x^{*}\leftarrow x^{tmp}$12:   **end if**13:  **end if**14: **end for**15: t←t+116:**end while**

### 5.2. Overall Process

Algorithm 2 shows the overall process of the proposed method. In Line 1, the bat population is initialized using a greedy method that will be described in Section 5.2.1 and the initial population is evaluated to find the best couple (Line 2). After that, the vectors of bats are updated through an iterative process (Lines 3–16). Note that the object function *f* in Lines 8 and 10 is equivalent to Equation (4). Thus the position vector x for a bat couple should be transformed into the sensing and relay node sets, $S_{s}$ and $S_{r}$, before being given to the object function *f* in actual.

#### 5.2.1. Population Initialization

First, the proposed method initializes frequency vector f, velocity vector v and position vector x for each couple. Like the usual BA, f and v of all bats are initialized to zero vectors. On the other hand, the position vector x is initialized by a greedy method, while most previous researches randomly decide the initial position. This is because a greedy method causes the algorithm to converge to the optimal solution faster than the random initialization method.

Algorithm 3 presents how to initialize the bat position. $N_{c}$ is the number of bat couples; and $x^{c}$ denotes the position vector of the *c*-th couple, which consists of two position vectors $x_{B_{s}}^{c}$ and $x_{B_{r}}^{c}$ for bat $B_{s}$ and bat $B_{r}$. To initialize $x^{c}$, $x_{B_{s}}^{c}$ is initialized first using a greedy method. Given a set of PoIs U={1,2,…,m} that have not been covered yet, the following steps are repeated until all PoIs are covered (U=ϕ):

(1) For each uncovered PoI *j* in *U*, one sensor node is randomly selected to be activated according to the following probability of each node *i*:
  (12) $$p_{i}\left(j\right)=\frac{\lambda_{i}\left(j\right)}{\sum_{n\in N}^{}\lambda_{n}\left(j\right)}$$
  where *N* is the set of sensor nodes.
(2) Any node being selected, then the corresponding element in the position vector $x_{B_{s}}^{c}$ is set to 1.
(3) PoIs whose detection probability exceeds the threshold value by activating a new sensor node in the previous step are removed from *U*.
(4) If U≠ϕ, return to the first step.

**Algorithm 3** Initialization of the bat position.1:**for***c* from 1 to $N_{c}$ **do**2: U={1,2,…,m} **// uncovered PoI list**3: **while** U≠ϕ **do**4:  **for** j∈U **do**5:   i←SelectNode(j)6:   $x_{B_{s},i}^{c}\leftarrow 1$7:   $C\leftarrow CoveredPoISet(x_{B_{s},i}^{c})$8:   U←U−C9:  **end for**10: **end while**11: $FindRelayNode(x_{B_{r}}^{c})$ // using a Steiner tree algorithm12:**end for**

After the $B_{s}$ initialization, the position vector $B_{r}$ to find relay nodes is initialized using a Steiner tree algorithm. In our Steiner tree, sensing nodes and relay nodes have the role of terminal vertices and Steiner vertices, respectively.

In order to construct a Steiner tree, this paper adopts the well-known heuristic method in [43] which uses the minimum spanning tree:

(1) First, make a graph G(V,E) where *V* is the set of all sensor nodes and *E* is the set of edges between two nodes that can directly communicate with each other.
(2) Then, construct a metric closure *M* of *G*, which is a complete graph with edge weights equal to the shortest distances in *G* [44].
(3) Construct a subgraph *H* of *M* induced by only the sensing nodes.
(4) Find a minimum spanning tree *T* of *H*.
(5) Construct a Steiner tree $T_{s}$ from *T* by replacing each edge with the corresponding shortest path in *G*.

According to [45,46], constructing a metric closure is the most time-consuming work. However, this step can be performed regardless of the specific terminal node set, so the proposed method needs to construct the metric closure only when sensor nodes are initially deployed or some sensor nodes run out of battery.

#### 5.2.2. Iterative Process

Lines 3–16 of Algorithm 2 are the main part that updates the bat position vector iteratively to find the optimal solution of the function *f*. $N_{t}$ and $N_{c}$ denote the allowed maximum number of iterations and the number of bat couples.

The procedure for each bat couple in one iteration is as follows:

(1) Slightly adjust the frequency $f^{c}$ and the velocity $v^{c}$ for bat couple *c*.
(2) Update $x^{tmp}$ using Algorithm 4.
(3) Evaluate $x^{tmp}$ based on Equation (4).
(4) If $x^{tmp}$ is better than previous position $x^{c}$ and has a valid position that can cover all PoIs and guarantee connectivity, then update $x^{c}$ with $x^{tmp}$.
(5) If $x^{tmp}$ is better than even the best position $x^{*}$ across all couples, then update $x^{*}$ with $x^{tmp}$, too.

In the first step, the velocity vector is updated before the position vector using the following equation:

(13) $$v_{b}^{c}\left(t+1\right)=v_{b}^{c}\left(t\right)+\left(x_{b}^{c}-x_{b}^{*}\right)f_{b}^{c},\;b\in \left\{B_{s},B_{r}\right\},$$

where $v_{b}^{c}\left(t\right)$ is the velocity of couple *c* at the *t*-th iteration and $f_{b}^{c}$ is the frequency vector. For simplicity, we set $F_{min}$ to 0 in Equation (8), thus frequency value is uniformly assigned in $\left[0,F_{max}\right]$.
**Algorithm 4** Position update.1:**for***i* from 1 to $N_{s}$ **do**2: **if** $\beta <r_{p}$ **then**3:  $x_{b,i}^{tmp}\leftarrow x_{b,i}^{*}$4: **else**5:  **if** $\rho <T(v_{b,i}^{c}\left(t\right))$ **then**6:   $x_{b,i}^{tmp}\leftarrow {\left(x_{b,i}^{c}\left(t\right)\right)}^{-1}$7:   $v_{b,i}^{c}\left(t\right)\leftarrow v_{b,i}^{c}\left(t\right)/2$8:  **else**9:   $x_{b,i}^{tmp}\leftarrow \left(x_{b,i}^{c}\left(t\right)\right)$10:  **end if**11: **end if**12:**end for**

In the second step, the position of each bat is updated for a new test. In the case of the conventional BA, of which the value in each dimension of the position vector is a real number, the position vector can be updated by simply using Equation (6). However, for the proposed BA where each element of the position vector has a binary value (0 or 1), a different method to update the position vector is required.

**Figure 4:**
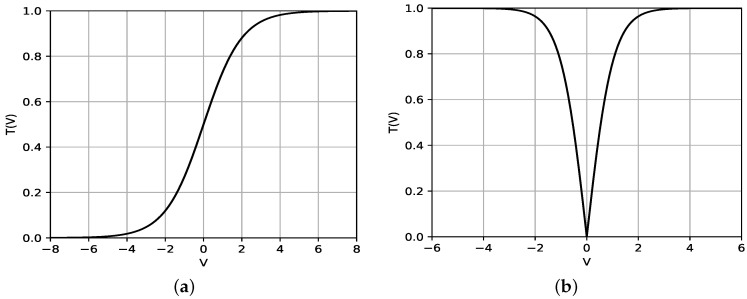
(**a**) S-shaped transfer function and (**b**) V-shaped transfer function.

There have been several approaches to this problem. One of them is to use a transfer function that firstly converts a value in each dimension of the velocity vector to a value in [0,1] as GSA [47], PSO [48] and BA [35] do. The transfer functions can be classified into two types: S-shaped and V-shaped. The S-shaped transfer functions, also known as sigmoid functions, were used for the conventional Binary NIAs [38,48,49,50]. Equation (14) is an example of the S-shaped function. According to the value of velocity *v*, T(v) changes like the shape of the alphabet *S* as shown in Figure 4a.

(14) $$T\left(v\right)=\frac{1}{1+e^{-v}}$$

The value T(v) is used to determine the position vector values. As an example, when $T(v_{i}\left(t\right))$ is the result of a transfer function for the *i*-th element value in the velocity vector at the *t*-th iteration, the corresponding element of the position vector, $x_{i}^{tmp}$, is updated according to the following rule:

(15) $$x_{i}^{tmp}=\left\{\begin{array}{c} 1,\;\mathrm{if}\;\rho <T(v_{i}\left(t\right))\\ 0,\;\mathrm{otherwise} \end{array}\right.$$

where ρ is a random number in [0,1].

On the other hand, the V-shaped function in [47] is represented as:

(16) $$T\left(v\right)=\left|tanh(v)\right|$$

As a result, T(v) has the shape of the alphabet *V* like Figure 4b. The value T(v) transformed by a V-shaped function is used as the basis for determining whether to keep the current binary value of the corresponding element in the position vector. When $x_{i}{\left(t\right)}^{-1}$ is the complement of $x_{i}\left(t\right)$ and ρ is a random number in [0,1], the *i*-th element value in the position vector is updated as follows:

(17) $$x_{i}^{tmp}=\left\{\begin{array}{c} x_{i}{\left(t\right)}^{-1},\;\mathrm{if}\;\rho <T\left(v_{i}\left(t\right)\right)\\ x_{i}\left(t\right),\;\mathrm{otherwise} \end{array}\right.$$

In fact, it is already known that the V-shaped transfer functions are usually better than the S-shaped transfer functions in terms of convergence rate and local minima avoidance [49,51]. Further, observing Figure 4b, we can see that the V-shaped function can depict bat’s fly better due to the following reasons:

- The velocity of zero means that the bat has arrived already at the optimal position. It does not have to move any more, so T(0) should be zero.
- A large difference of the velocity from zero means that the bat is far from the optimal position. As the absolute value of velocity increases, T(v) should also increase.

On the contrary, the S-shaped function does not have the features above, thus our method adopts the V-shaped transfer function.

In summary, when $v_{b,i}^{c}\left(t\right)$ is the *i*-th element in the velocity vector of bat *b* in bat couple *c* at the *t*-th iteration, the proposed method first computes the transfer function value as follows:

(18) $$T\left(v_{b,i}^{c}\left(t\right)\right)=\left|tanh(v_{b,i}^{c}\left(t\right))\right|$$

Then each element of the position vector $x_{b,i}^{tmp}$ is updated according to the following rule:

(19) $$x_{b,i}^{tmp}=\left\{\begin{array}{c} x_{b,i}^{c}{\left(t\right)}^{-1},\;\mathrm{if}\;\rho <T\left(v_{b,i}^{c}\left(t\right)\right)\\ x_{b,i}^{c}\left(t\right),\;\mathrm{otherwise} \end{array}\right.$$

Note that if the *i*-th dimension of the position vector is changed, the velocity of the *i*-th dimension is reduced by half (Line 7 in Algorithm 4). This prevents position vectors from oscillating too wide a range in the iteration step.

In the third to fifth steps of the procedure, the sink node checks if the new position is better than the previous one. If so, the validation is performed to check whether the new position covers all PoIs and ensures connectivity. Since the computational complexity of the cost function *f* is lower than the complexity of the validation, this function is performed prior to the validation. The valid new position replaces the previous position vector. If the position is better than the best position $x^{*}$, the best position is also replaced.

## 6. Simulation

We performed a simulation to compare the performance of the proposed method with others. The comparison methods were divided into two groups. The first group only considered the target coverage, assuming that all sensor nodes had sufficient communication range to transfer their data directly to the sink node. Thus Section 6.4 compared our method with the first group in terms of energy consumption and computation complexity to meet the required target coverage rate without considering the connectivity. On the other hand, the second group took account of the connectivity as well as the sensing coverage. In Section 6.5, each sensor node had a limited communication range, so multi-hop paths to the sink node were used depending on the distance between a sensor node to the sink. All simulations were performed on a PC with an Intel i5-7500 CPU and 8 GB of RAM, and were implemented using Python and NetworkX package [43]. The source codes are available in https://github.com/repushed.

**Figure 5:**
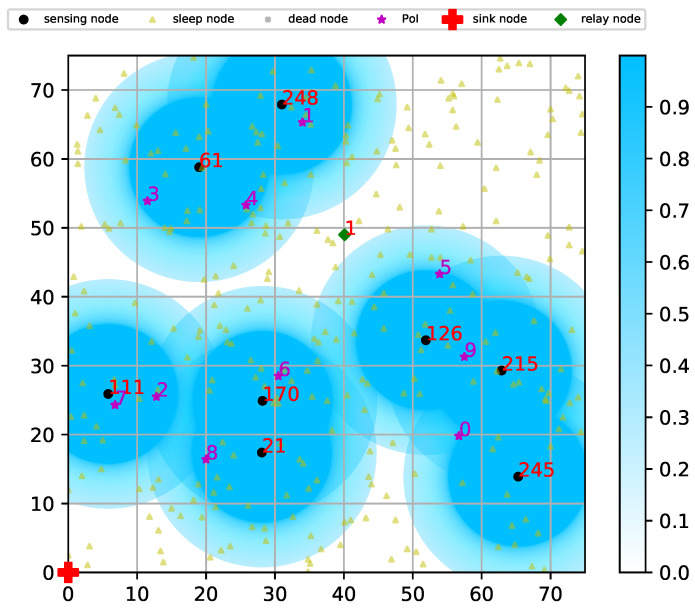
Depiction of a network at a timeslot.

Figure 5 depicts an example of a network status at a timeslot. The network with 75×75 m${}^{2}$ size consisted of 300 sensor nodes and 10 PoIs. The sensing parameters in Equation (2) were set as follows: $r_{s}=10$ m, $r_{u}=16.5$ m, k=0.5 and q=0.5. The sensing threshold ϵ=0.9 and the communication range of each sensor node was twice of the sensing range $r_{u}$, or 33 m. First, PoIs were denoted by the purple stars. Then nodes activated to sense PoIs are denoted by the black circles, whereas sleeping nodes were denoted by the yellow triangles. The numbers next to the active nodes and PoIs indicate their ID. The blue circles surrounding each sensing node illustrate the sensing range mentioned in Equation (2). Finally, the intensity of blue color means the cumulative sensing probability of each position.

As mentioned above, we focused on only the coverage in Section 6.4, assuming all sensor nodes could directly communicate with the sink node. However, in Section 6.5, we set the communication range to double of $r_{u}$, although it is not illustrated in Figure 5. Due to the limited communication range, sensing nodes 61 and 248 could not communicate with any other sensing nodes, so node 1 was activated to play the role of a relay node (denoted by the green diamond). As a result, sensing node 61 and 248 could deliver its data to the sink at (0, 0) via a multi-hop path with the help of relay node 1 and other sensing nodes.

### 6.1. Optimal Parameters in Proposed Method

First of all, we had to determine the number of iterations ($N_{t}$) and the number of bat couples ($N_{c}$) for the proposed method, because the performance of the sensor activation method is affected by them. In order to find out the optimal $N_{t}$ and $N_{c}$, we performed an experiment. In the experiment, the sensing parameters in Equation (2) were set as follows: $r_{s}=10$ m, $r_{u}=16.5$ m, k=0.5 and q=0.5. The sensing threshold ϵ=0.9 and the communication range of each sensor node was twice of the sensing range $r_{u}$, or 33 m. Without loss of generality, all sensor nodes initially had 30 units of energy; and were assumed to use one unit and two units of energy for sensing and communication for one timeslot. Therefore, a sensing node consumed 3 units of energy per timeslot since it had to perform sensing and communication at the same time. A relay node just consumed 2 units of energy for communication. For the sake of simplicity, the energy consumption of sleeping nodes was not taken into account. This assumption is identical to them in the simulation for PSCA [29] and is also used in our other experiments (Section 6.2, Section 6.3 and Section 6.5). A total of 300 sensor nodes and 20 PoIs were uniformly deployed in the 75×75 m${}^{2}$ network and this setting is identical to the last scenario in Section 6.5. Because the scenario had the largest scale on the number of sensors and PoIs, this was the most complex one among all scenarios in our experiments. We used this scenario to find optimal values of $N_{c}$ and $N_{t}$. The experiment was performed 30 times.

**Figure 6:**
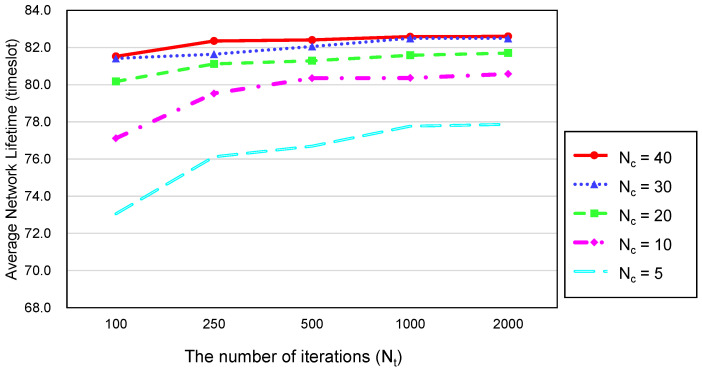
Average network lifetime against $N_{c}$ and $N_{t}$.

**Figure 7:**
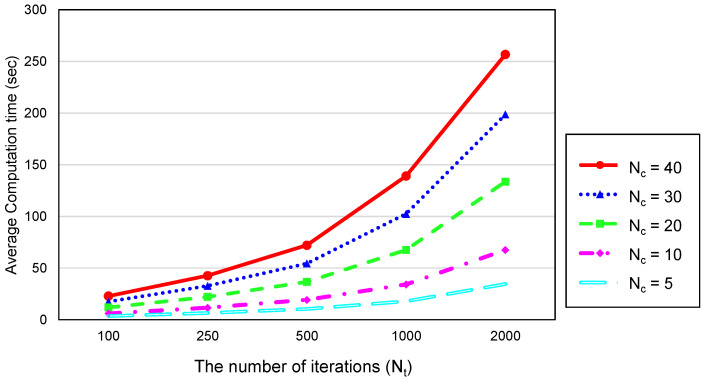
Average computation time against $N_{c}$ and $N_{t}$.

Figure 6 shows the network lifetime against the number of bat couples $N_{c}$ and the number of iterations $N_{t}$. The network lifetime was defined as the timeslots passed until there was no set of active nodes that covered all PoIs or guaranteed the connectivity between the sink and sensing nodes. As $N_{c}$ and $N_{t}$ increased, the average lifetime also increased. However, the network lifetime converged to 78 timeslots, no longer increasing after the number of bat couples $N_{c}$ is 30 and the number of iterations $N_{t}$ is 1000. As shown in Figure 7 which illustrates the average computation time, the larger $N_{c}$ and $N_{t}$, the more the computation time of the proposed method. Therefore, we adopted 30 and 1000 for $N_{c}$ and $N_{t}$ respectively.

### 6.2. Weight Values for the Cost Function

In Equation (4), the proposed method finds the solution of the cost function *f*, considering the residual energy of each sensor. How much the residual energy affects the solution of *f* depends on the value of κ in Equation (5). So, the experiment was performed to find the optimal value of κ. According to the result of the previous experiment, $N_{t}$ and $N_{c}$ are fixed to 1000 and 30, respectively.

**Figure 8:**
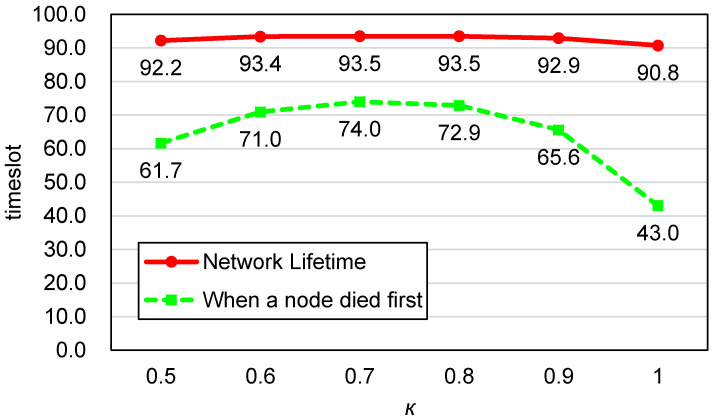
Average network lifetime against κ in Equation (5).

Figure 8 shows the average network lifetime and the average number of timeslots passed until a node died first while the value of κ was from 0.5 to 1 with 0.1 steps. When the sensor residual energy was not considered (κ=1), the performance was the worst, while it was the best when κ was 0.7. This result substantiates that the residual energy information is useful in extending the time taken to the first depletion of node energy. This can be explained as follows: If the residual energy was not considered, the cost function in Equation (4) would give the minimum value when the minimum number of sensors were activated. Thus, a small number of sensors were given all the burden, leading to the energy depletion of the sensors. As a result, as the time grew, the number of alive sensors quickly decreased, resulting in the inefficient sensor sets $S_{s}$ and $S_{r}$ to cover all PoIs. On the other hand, the consideration of residual energy lengthened the lifetime of each sensor, thus diverse sensor sets could be found to cover PoIs, resulting in a higher chance to find the more efficient sensor sets $S_{s}$ and $S_{r}$.

### 6.3. Necessity of a New Bat Algorithm

**Figure 9:**
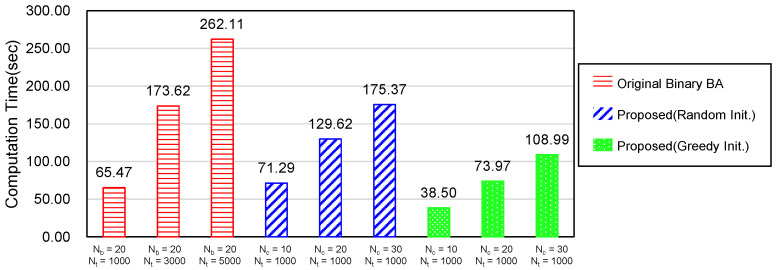
Average computation time according to the versions of Binary Bat Algorithm.

**Figure 10:**
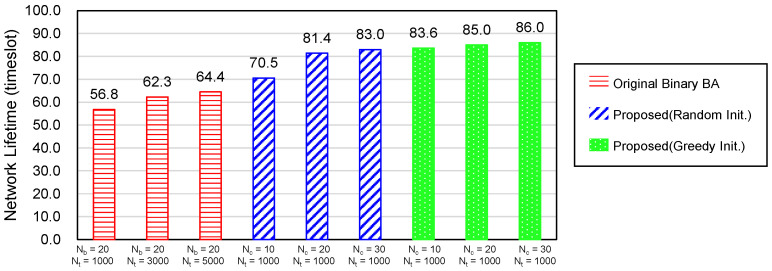
Average network lifetime according to the versions of Binary Bat Algorithm.

This chapter discusses why we proposed a new BA although the binary bat algorithm (BBA) [35] uses binary values for the position vector like the our method. First, we attempted to apply the BBA to the sensor coverage problem, but the performance was not good enough. Figure 9 and Figure 10 illustrate the average computation time per timeslot and the network lifetime of the BBA and our proposed method. The leftmost three red bars in the both graphs show the performance of the BBA. The number of bats $N_{b}$ was set to 20 because the recommended value of $N_{b}$ is 10 to 20 according to [35] and the number of iterations $N_{t}=$ 1000, 3000, or 5000. All other parameters are the same as in Section 6.1 and the simulation was performed 30 times. The result shows that the BBA could not find the optimal solution even with a very large number of iterations, $N_{t}=5000$. On the contrary, our scheme, denoted by the rightmost three green bars, approached to the optimal value much more quickly with only $N_{t}=1000$ and the number of bat couples $N_{c}=$ 10, 20 or 30. This proves that the concept of bat couple and the greedy initialization method in the proposed scheme can make a big difference from the normal BBA.

Besides the performance, we had another reason to develop a new BA. While each sensor in the normal BBA can have one of two states (active and sleeping), we needed a scheme where a sensor can have one out of three states, relaying, sensing, and sleeping.

When evaluating the performance according to the initialization method in our method, the greedy initialization reduced the computation time to about 47.5–57.5% against the random initialization depending on the number of bat couples. As to the network lifetime, the greedy method also slightly outperformed the random initialization. Particularly, the performance gap increased as the number of bat couples decreased. This means that the smaller number of bats with the random initial position may not have found a solution close to the optimal one. Based on these results, the greedy initialization was utilized for all our experiments, unless otherwise stated.

### 6.4. First Comparison Experiment: Target Coverage Only

The experiment in this section considered only target sensing coverage by making the proposed method just skip Line 11 in Algorithm 3. Sensor nodes and PoIs were randomly deployed in the 10×10 m${}^{2}$ area. Initially, all sensor nodes had the same amount of energy, which can be used to sense a PoI and report it to the sink 10 times. We ignored the energy consumed by nodes in the sleeping mode. The sensing parameters were set as follows: $r_{s}=1.5$ m, $r_{u}=6$ m, k=0.5, and q=0.5. This is the same as in the JOA simulation [21] for the fair comparison. We compared the performance with three NIA methods such as PSO [19], ACO [19] and JOA [21], which take account of only the target coverage problem. Simulation parameters for the comparison methods came from the previous literature [21], and the parameters for the proposed BA were set as follows: the number of iterations $N_{t}=1000$, the number of bat couples $N_{c}=20$ or 30, and the pulse rate $r_{p}=0.5$. There were six different scenarios according to the number of sensor nodes and PoIs, which is shown in Table 1. We performed the simulation 30 times for each scenario.

Table 2 compares the average lifetime of sensor nodes for each method, and especially the last two columns labeled BA are the proposed method with different number of bat couples, 20 or 30. Our method was superior or almost similar to the others in most cases. With Scenarios 3, 5, and 6, PSO was better than the proposed method, but PSO has the significant scalability problem, which will be explained below soon. It is noteworthy that the lifetime rose faster than the rate at the number of sensor nodes increases. This is related to the probabilistic sensing feature used in this paper. As in Equation (3), in order to cover one PoI in the probabilistic sensing model, the cumulative probability of all sensing nodes for the PoI should exceed the threshold probability.

In addition, the Friedman test [52] was used to assess whether there are any statistically significant differences of the performance among the algorithms. The test can be used primarily to approve the null hypothesis that multiple group measures have the same variance for a specific required significance level. *p*-value, calculated by the Friedman test, is used to determine whether null hypothesis is true or not. If *p* is smaller than a pre-defined significance level α, null hypothesis is rejected, otherwise approved [53]. Our null hypothesis $H_{0}$ is that all algorithms used in the experiment have the same performance in terms of network lifetime, and alternative hypothesis $H_{1}$ is that the algorithms of the experiment have distinct performance differences between them. Table 3 shows the average ranking of network lifetime and *p*-value computed by the Friedman test for each scenario. Because all the *p*-values are less than 0.05, the null hypothesis $H_{0}$ is rejected at the significance level 5% (α=0.05) and we can conclude that the algorithms have distinct performance differences between them.

As the number of sensor nodes decreased, the average distance from a sensor node to a PoI increased, so the detection probability λ by each sensor for any PoI exponentially decreased. Therefore, relatively more sensors should be activated to meet the required cumulative detection probability, when the number of sensor nodes is small.

Table 4 shows the average computation time in one timeslot. The proposed method outperformed all the other methods in most cases. The only exception was JOA when there were 100 nodes and $N_{c}=30$ (Scenarios 1 and 4). However, if we reduced the number of bat couples to 20, the proposed method was faster than even JOA. JOA activated all sensor nodes in the beginning, then deactivated them one by one through an iterative process while the required coverage was satisfied. Therefore, as the number of sensor nodes increased, much more time was needed in selecting the sensor nodes to be deactivated. Meanwhile, as shown in Table 2 and Table 3, PSO was the best in terms of node average lifetime, but it required about 20 times longer computation time than our method. This is because PSO randomly initialized the population. Since the proposed method initialized the bat couples using a greedy method, it could quickly find the optimal solution with relatively few iterations. Conclusively, considering both the scalability and the energy efficiency, the proposed method was the best choice.

### 6.5. Second Comparison Experiment: Target Coverage and Network Connectivity

Unlike the methods in Section 6.4, PSCA [29] and LoCQAl [27] guarantee the connectivity to sink as well as target coverage. Table 5 shows a total of 12 scenarios with various numbers of sensor nodes and PoIs in two areas of different sizes. We performed the simulation 30 times for each scenario. Like the Section 6.1, the sensing parameters were set as $r_{s}=10\;m$, $r_{u}=16.5\;m$, k=0.5, and q=0.5. The communication range was 33m, the double of the sensing range. The initial energy of each sensor node was 30 units. A sensing node consumed 3 units of energy per timeslot and a relay node consumed 2 units of energy. This assumption was identical to the PSCA experiment [29] for the fair comparison.

Table 6 shows the average lifetime of sensor nodes for each scenario. Table 7 presents the average ranking of network lifetime for each scenario. The *p*-values by the Friedman test were very small (p<<0.05). Therefore, the test shows that there were distinct performance differences among the algorithms in terms of network lifetime. In most scenarios, the proposed method had the lifetime of 10% longer than LoCQAI and PSCA. First, LoCQAI had the worst performance because it selects nodes according to their priorities. Therefore, some sensor nodes on critical points may be always turned on to provide connectivity. Meanwhile, the process of PSCA was divided into two steps: After selecting sensing nodes, it found relay nodes using a Steiner tree algorithm separately. This separation of the two steps may have caused unnecessary sensor nodes to be activated. Moreover, PSCA did not take into account the remaining energy of sensor nodes. As to the proposed method, the network lifetime improved as the number of bat couples ($N_{c}$) increased. This is because the larger the number of bat couples, the more likely it could find a global solution without falling into local optima thanks to the various initial positions of the couples. The proposed method used a Steiner tree to find relay nodes, similar to PSCA. However, the proposed method could find the better solution, because it found the relay nodes and sensing nodes simultaneously through an iterative process and it considered residual energy of each sensor node.

Table 8 shows the computation time for each scenario. LoCQAl was the fastest because it had no highly complex iterative process unlike the proposed method. On the other hand, although PSCA was generally faster than the proposed method, its computation time increased significantly as the number of nodes increased or the size of the network decreased. This is because PSCA utilized a recursive DFS algorithm to find the candidate cover set (CCS) for each PoI. In the DFS operation, the cumulative detection probability for each PoI by all sensor nodes was calculated. The time complexity of a usual DFS is known as O(V+E), where *V* and *E* are the numbers of nodes and edges. In PSCA, the DFS was performed for every PoI, so the number of PoIs, *T*, should be multiplied to the time complexity, resulting in O[T(V+E)]. Here, *E* was determined by the number of neighbors of each node: In the worst case, $\left|E\right|=\frac{V(V-1)}{2}$ in a complete undirected graph. Thus, the actual computation time increased as (1) the number of PoIs increased, (2) the number of sensor nodes increased, or (3) the network size decreased because if nodes were deployed in smaller area, the number of neighbors would increase. On the contrary, the computation time of the proposed method increased linearly with the number of nodes as shown in Table 8. Therefore, if there were many nodes in a small network, the proposed method would be the best choice.

Compared with LoCQAl, the proposed method needed much higher computation time. However, the proposed method showed the longer network lifetime. Especially, the more complicated the environment (e.g., more sensors and targets), the larger the performance gap was. The computation time depended on the hardware capability of the sink, and the network lifetime was related to the energy resource of sensor nodes. In WSNs, sensors usually have limited energy while the sink is composed of powerful hardware. Furthermore, considering that a large number of sensors exist in a network area, energy saving of sensor nodes is more important than short computation time of sink nodes.

## 7. Conclusions

We suggested an energy-efficient sensing and communication method using the bat algorithm to guarantee connectivity to sink as well as target coverage. In the simulation using the probabilistic sensing model for practicality, the proposed method converged to the optimal solution faster than the methods based on other nature-inspired algorithms such as PSO, ACO, and JOA. Compared to LoCQAl and PSCA, although the proposed method required more computations in most network environments, the network lifetime could be improved by more than 10%. Furthermore, the proposed method has the scalability, so it showed faster computation speed compared to PSCA, particularly when there were many nodes in small size of network area.

In future work, we will enhance the proposed method to consider routing protocols simultaneously. Our method currently considers only the connectivity from active sensor nodes to the sink node in selecting relay nodes, but the energy consumption of each node may depend on the number of communication paths passing through it. Therefore, if routing algorithms are taken into account together, more energy-efficient sensing and communication methods can be designed.

## Figures and Tables

**Table 1:** Scenarios for the first experiment.

#	Number of Sensor Nodes	Number of PoIs
	($N_{s}$)	($N_{p}$)
1	100	10
2	150	10
3	200	10
4	100	30
5	150	30
6	200	30

**Table 2:** Average lifetime in the first experiment.

#	Average Lifetime (Timeslot)				
	ACO	PSO	JOA	BA ($N_{c}=20$)	BA ($N_{c}=30$)
1	50.8	50.9	51.6	51.9	52.4
2	79.7	83.5	81.3	81.9	83.2
3	113.8	120.4	117.6	117.7	119.0
4	39.8	41.5	40.1	42.5	42.7
5	58.8	65.4	60.6	63.8	65.6
6	82.3	91.8	82.4	88.4	89.4

**Table 3:** Ranking by Friedman test for each scenario in the first experiment.

#	Rank by Friedman Test					*p*-Value
	ACO	PSO	JOA	BA ($N_{c}=20$)	BA ($N_{c}=30$)	
1	3.96	3.46	3.06	2.60	1.92	$5.965\times e^{-5}$
2	4.46	2.02	3.38	3.10	1.86	$2.190\times e^{-10}$
3	4.88	1.58	3.24	3.24	2.06	$2.023\times e^{-13}$
4	4.42	2.74	4.08	2.00	1.76	$8.087\times e^{-12}$
5	4.74	1.46	4.19	2.63	1.98	$3.253\times e^{-30}$
6	4.63	1.27	4.32	2.80	1.98	$1.275\times e^{-19}$

**Table 4:** Average computation time in the first experiment.

#	Average Computation Time (Sec)				
	ACO	PSO	JOA	BA ($N_{c}=20$)	BA ($N_{c}=30$)
1	20.06	155.59	9.32	8.76	11.80
2	30.96	257.97	19.35	12.88	19.22
3	47.16	333.42	34.78	16.81	26.26
4	23.22	192.82	11.23	9.17	13.99
5	35.03	304.67	22.43	14.13	21.79
6	52.54	415.39	38.78	20.90	32.64

**Table 5:** Scenarios for the second experiment.

#	Number of Sensor Nodes	Number of PoIs	Network Size
	($N_{s}$)	($N_{p}$)	(m${}^{2}$)
1	100	10	50×50
2	100	20	
3	200	10	
4	200	20	
5	300	10	
6	300	20	
7	100	10	75×75
8	100	20	
9	200	10	
10	200	20	
11	300	10	
12	300	20	

**Table 6:** Average lifetime in the second experiment.

#	Average Lifetime (Timeslot)				
	LoCQAl	PSCA	BA	BA	BA
			($N_{c}=10$)	($N_{c}=20$)	($N_{c}=30$)
1	41.6	53.8	62.1	62.08	62.3
2	32.3	42.2	45.4	46.5	46.7
3	97.1	135.2	145.8	148.3	149.4
4	81.7	112.3	125.0	126.4	127.3
5	136.6	190.1	211.5	214.8	216.0
6	122.7	168.4	186.1	188.4	189.9
7	21.2	23.3	25.5	25.6	25.6
8	15.0	16.0	17.9	18.1	18.3
9	55.4	64.6	67.8	68.6	68.7
10	38.6	44.4	48.8	49.9	50.0
11	79.9	94.6	97.6	99.4	100.1
12	67.7	80.0	87.6	89.3	90.0

**Table 7:** Ranking by Friedman test for each scenario in the second experiment.

#	Rank by Friedman Test					*p*-Value
	LoCQAl	PSCA	BA ($N_{c}=10$)	BA ($N_{c}=20$)	BA ($N_{c}=30$)	
1	4.93	3.70	2.74	1.76	1.87	$2.119\times e^{-13}$
2	4.75	3.20	2.93	2.11	2.00	$9.994\times e^{-9}$
3	5.00	3.83	2.98	1.90	1.29	$2.942\times e^{-15}$
4	5.00	3.94	2.67	2.03	1.36	$1.016\times e^{-12}$
5	5.00	3.68	2.89	1.92	1.50	$2.916\times e^{-12}$
6	5.00	3.76	2.95	1.87	1.42	$5.258\times e^{-13}$
7	3.85	3.45	2.6	2.55	2.55	$1.303\times e^{-3}$
8	3.83	3.57	2.78	2.55	2.27	$2.370\times e^{-4}$
9	4.96	3.30	2.67	2.11	1.96	$4.363\times e^{-13}$
10	4.83	3.63	2.73	1.98	1.83	$3.209\times e^{-12}$
11	4.96	3.2	3.17	2.15	1.52	$8.377\times e^{-13}$
12	4.96	3.83	2.70	2.02	1.50	$9.222\times e^{-15}$

**Table 8:** Average computation time in the second experiment.

#	Average Computation Time (s)				
	LoCQAl	PSCA	BA ($N_{c}=10$)	BA ($N_{c}=20$)	BA ($N_{c}=30$)
1	0.03	0.95	9.85	18.75	28.30
2	0.05	1.07	9.95	18.90	28.18
3	0.07	11.10	19.33	38.21	56.89
4	0.12	17.41	22.71	45.12	62.18
5	0.23	103.84	36.91	66.57	93.01
6	0.29	208.47	30.55	60.97	90.19
7	0.04	0.25	10.90	20.39	31.19
8	0.03	0.26	9.88	19.50	30.16
9	0.06	1.90	19.45	38.40	57.65
10	0.11	2.26	20.71	40.77	58.78
11	0.21	8.58	31.40	60.48	90.37
12	0.23	12.53	31.48	61.68	102.94
